# Mesoporous Acidic Catalysts Synthesis from Dual-Stage and Rising Co-Current Gasification Char: Application for FAME Production from Waste Cooking Oil

**DOI:** 10.3390/ma13040871

**Published:** 2020-02-15

**Authors:** Junaid Ahmad, Umer Rashid, Francesco Patuzzi, Nahla Alamoodi, Thomas Shean Yaw Choong, Soroush Soltani, Chawalit Ngamcharussrivichai, Imededdine Arbi Nehdi, Marco Baratieri

**Affiliations:** 1 Faculty of Science and Technology, Free University of Bolzano, Piazza Universita 5, 39100 Bolzano, Italy; Francesco.patuzzi@unibz.it (F.P.); Marco.baratieri@unibz.it (M.B.); 2 Department of Chemical Engineering, Khalifa University, Abu Dhabi 00000, UAE; Nahla.alamoodi@ku.ac.ae; 3 Institute of Advanced Technology, Universiti Putra Malaysia, 43400 UPM Serdang, Selangor, Malaysia; 4 Department of Chemical and Environmental Engineering, Faculty of Engineering, Universiti Putra Malaysia, Serdang 43400, Malaysia; csthomas@upm.edu.my (T.S.Y.C.); soroush.soltaani@gmail.com (S.S.); 5 Center of Excellence in Catalysis for Bioenergy and Renewable Chemicals (CBRC), Faculty of Science, Chulalongkorn University, Bangkok 10330, Thailand; Chawalit.Ng@chula.ac.th; 6 Chemistry Department, College of Science, King Saud University, Riyadh 11451, Saudi Arabia; inahdi@ksu.edu.sa; 7 Laboratoire de Recherche LR18ES08, Chemistry Department, Science College, Tunis El Manar University, Tunis 2092, Tunisia

**Keywords:** dual-stage gasification char, rising co-current gasification char, post-sulfonation, characterization, transesterification, biodiesel

## Abstract

The main purpose of this work is to investigate the application options of the char produced from gasification plants. Two promising mesoporous acidic catalysts were synthesized using char as a support material. Two char samples were collected from either a dual-stage or a rising co-current biomass gasification plant. The catalysts produced from both gasification char samples were characterized for their physiochemical and morphological properties using N_2_ physorption measurement, total acidity evaluation through TPD-NH_3_, functional groups analysis by FT-IR, and morphology determination via FESEM. Results revealed that the dual-stage char-derived mesoporous catalyst (DSC-SO_4_) with higher specific surface area and acidic properties provided higher catalytic activity for fatty acid methyl esters (FAME) production from waste cooking oil (WCO) than the mesoporous catalyst obtained from char produced by rising co-current gasification (RCC-SO_4_). Furthermore, the effects of methanol/oil molar ratio (3:1–15:1), catalyst concentration (1–5 wt.% of oil), and reaction time (30–150 min) were studied while keeping the transesterification temperature constant at 65 °C. The optimal reaction conditions for the transesterification of WCO were 4 wt.% catalyst concentration, 12:1 methanol/oil molar ratio, and 90 min operating time. The optimized reaction conditions resulted in FAME conversions of 97% and 83% over DSC-SO_4_ and RCC-SO_4_ catalysts, respectively. The char-based catalysts show excellent reusability, since they could be reused six times without any modification.

## 1. Introduction

Fatty acid methyl esters (FAME), also known as biodiesel, can be produced by the transesterification/esterification process, a chemical reaction of fat/oil and oil-derived fatty acids with alcohol in the presence of a catalyst [1]. The choice of catalyst depends on the feedstock nature. There are a variety of acidic catalysts that have been explored to produce biodiesel so far, e.g. ion-exchange resin Amberlyst-15 [2], sulfated zirconia alumina [3], sulfonated mesoporous zinc aluminate, polymeric mesoporous zinc oxide [4], and Zn-substituted waste-eggshell-derived CaO nanocatalyst [5]. The main disadvantage of metallic-based catalysts is that their production cost is very high, which is considered one of the main hindrances in their application on industrial scale. Conversely, carbon-based catalysts are more feasible on an industrial scale for FAME production, owing to their cost-effectiveness and environmentally friendly nature.

The biomass-derived chars are excellent candidates as inexpensive sources of carbonaceous catalyst supports. However, there is a considerable amount of char being produced as a by-product worldwide, which is considered as waste. There is a need to utilize them effectively or dispose of them safely. Char shows excellent physiochemical properties, i.e. porous structure, high specific surface area, and high chemical and thermal stability. These characteristics make it increasingly applicable for catalytic reactions or as a catalyst support instead of metallic-based support. Besides, it is inexpensive, biodegradable, and naturally contains trace elements [6,7,8]. Recently, waste and biomass-derived waste products have been used for the preparation of catalyst supports instead of using metallic supports [8]. Carbon-based catalysts possess unique characteristics such as high specific surface area, flexible pore size, and high thermal stability, which makes them more attractive as compared to metallic support-based catalysts [9,10,11,12].

Gasification is a process of transforming the variety of feedstock into gaseous products [13] by reacting biomass at high temperatures (>700 °C) in the presence of oxygen and/or steam. The subsequent gas mixture is named syngas, whereas tar and char are achieved as by-products. Among all the thermochemical processes (combustion, slow/fast pyrolysis, torrefaction, and gasification) that produce char as a byproduct, char yield is lower in the gasification process. Gasification technology is gaining attention around the world for the transformation of solid biomass into potential renewable energy.

The exploration of waste cooking oil (WCO) potential and its utilization as a raw material for FAME production have their own merits and demerits. It is known that a high reaction temperature speeds up cracking of triglycerides. Contrarily, a high reaction temperature in the presence of water molecules may increase free fatty acid (FFA) contents, resulting in high viscosity via soap formation [14]. To avoid these hurdles, a vast survey is required to achieve higher biodiesel production yields. One possible approach is improving the hydrophobicity of the catalyst, which reduces water adsorption onto the active sites of catalysts [15]. Post-acid treatment is another favorable technique, which modifies the hydrophobicity of the active catalyst sites. Through the esterification reaction, a hydrophobic surface catalyst prevents the presence of moisture on the active sites. Commonly, hydrophobicity is a key property to prevent the decomposition of the catalyst during the catalytic reaction. Through the post-sulfonation treatment, sulfonic groups (SO_3_H) attach on the surface of prepared samples and transform the catalyst’s nature [16].

Herein, sulfonated mesoporous catalysts were prepared using a dual-stage (DSC-SO_4_) or a rising co-current (RCC-SO_4_) biomass gasification plant and used for the transesterification of WCO. The focus of this paper is to determine how chars obtained from different gasification technologies affect the synthesis of mesoporous sulfonated carbon catalysts. Furthermore, the pivotal process parameters of catalyst amount, methanol/WCO molar ratio, and reaction time of the transesterification reaction were optimized using a batch system. Finally, catalyst reusability was studied by using the optimal transesterification conditions.

## 2. Materials and Methods

### 2.1. Materials

The raw char samples were procured from biomass gasification plants located in the South Tyrol region, Italy. The first char (DSC) was produced in a dual-stage gasifier. The plant has nominal electric power and thermal power outputs of 50 kWel and 80 kWth, respectively. The second char (RCC) was produced in a rising co-current gasifier that uses pellets as feedstock and air as a gasifying agent. The plant has nominal electric power and thermal power output ranges of 180–190 kWel and 220–240 kWth, respectively. The cafeteria of the Free University of Bolzano, Bolzano, Italy, supplied WCO. The chemicals and reagents, such as methanol, hexane, acetone, and concentrated sulfuric acid (98%) were bought from Merck (Kenilworth, NJ, USA). All these reagents were analytical-grade and utilized as received without further processing.

### 2.2. Preparation of DSC-SO_4_ and RCC-SO_4_ Catalysts

The mesoporous char-supported solid acid catalysts were prepared as proposed by Dehkhoda et al. [17]. Briefly, 10 g of each raw char was blended with sulfuric acid (100 mL). The resultant mixture was then heated at 150 °C for 6 h in a closed-cup autoclave. The obtained sulfated mesoporous char catalysts were washed with water and hexane until the water from washing became neutral. The prepared sulfonated mesoporous catalysts from dual-stage gasification (DSC-SO_4_) and co-current gasification (RCC--SO4) of char were dried at 70 °C and stored for further characterization.

### 2.3. Produced Catalysts Characterization

The textural, physiochemical, and morphological features of the raw chars and char-based catalysts were determined by employing a variety of characterization instruments. The Brunauer, Emmett, and Teller method was used to evaluate the specific surface area (S_BET_), whereas N_2_ physisorption measurement using a 3 Flux micromeritics, Norcross, GA, USA, was used to determine the pore size and pore distribution. Before the analysis, each sample was degassed externally at 350 °C for 12 h beneath a constant flow of N_2_ to purify the sample from moisture. The adsorption–desorption experiment was carried out using N_2_ at −196 °C. The acid density (TPD-NH_3_) of the samples was measured using Thermo Finnigan TPDRO 1100 (Hampton, NH, USA), which was attached to the thermal conductivity detector (TCD). For the analysis, 500 mg of catalyst sample was treated hydrothermally under the inert atmosphere of argon at 150 °C in order to adsorb the water from the atmosphere and additionally to remove the impurities. When the raw chars and char-based catalysts were cooled down, each sample was treated again with ammonia gas at a flow rate of 150 mL/min for one hour. Lastly, the treated sample was heated at 15 °C/min heating rate with a 50 mL/min helium flow up to 900 °C, and the TCD detector was employed to calculate the total adsorbed ammonia concentration on the samples. Consequently, the functional groups of char samples and prepared catalysts were examined by using Fourier-transform infrared (FT-IR) spectroscopy. An FT-IR spectrometer (Thermo Nicolet 5ZDX, Waltham, MA, USA) was used to investigate the transmittance between 400 cm^−1^ and 4000 cm^−1^ with a resolution of 4 cm^−1^. Field emission scanning electron microscopy (FE-SEM; FEI Nova NANAOSEM 230 microscope, Hillsboro, OR, USA) was used to examine the surface morphology of each sample.

### 2.4. Transesterification Reaction with Produced Catalysts

Before starting the transesterification reaction, the WCO was warmed at 110 °C for 60 min to attain a high FAME yield. Next, the catalytic activity was examined in the presence of char-based catalysts using a batch reactor system. Transesterification of WCO was performed in a 250 mL three-neck glass reactor attached to a condenser to re-condense the evaporated methanol. The reactor was dipped into a silicone oil bath that was placed on a hot plate and equipped with a magnetic stir and a thermocouple. Magnetic stirring ensured continuous mixing, while the silicon oil bath served to provide homogeneous heating throughout the reaction. The influence of the critical transesterification parameters, i.e., amount of catalyst (1–5 wt.%), methanol/WCO molar ratio (3:1–15:1), and reaction time (30–150 min), were then examined in separate studies, keeping the reaction temperature constant at 65 °C for all batches. After a specified time for each reaction, the obtained mixture was centrifuged using a high-speed centrifuge at 7000 rpm for 10 min to separate three phases: FAMEs, catalyst, and glycerol. Finally, the produced FAMEs were preserved for further analysis.

### 2.5. Reusability of the Catalyst

Catalytic stability is considered as one of the main properties of heterogeneous catalysts, which significantly determines the cost of production on an industrial scale. The reusability experiment was carried out under the obtained optimal transesterification conditions. After each run, the spent catalyst was centrifuged and splashed with hexane, followed by acetone washing to eliminate the oil, methanol, and glycerol particles stuck on the catalyst surface. After several rounds of washing, the recovered catalyst was dried overnight in an oven (110 °C) to be used for the next transesterification reaction.

### 2.6. Biodiesel Analysis

The FAME yield was calculated by means of GC-FID according to Dehkhoda et al. [17].

$$FAME\;Yield=\frac{\sum A\;-\;A_{meh}}{A_{meh}}\times \frac{C_{meh}x\;V_{meh}}{Wt}\times 100\%$$

where ∑*A* is the area under the FAME peaks. The terms *A_meh_*, *C_meh_*, *V_meh_*, and *W_t_* represent the peak area, concentration, and volume of methyl heptadecanoate and the mass of the FAME produced, respectively.

## 3. Results and Discussion

### 3.1. Characterization of DSC-SO_4_ and RCC-SO_4_ Catalysts

#### 3.1.1. Surface Area Analysis

**Figure 1:**
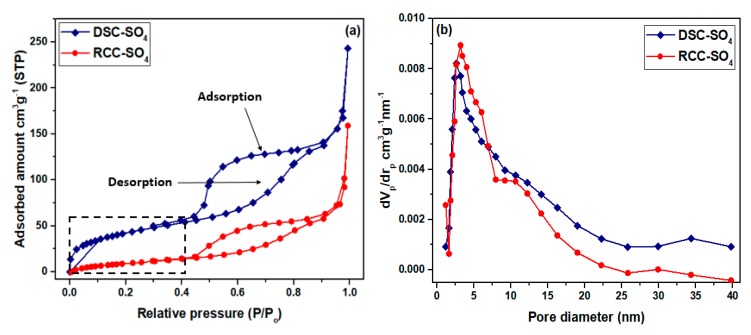
(**a**) N_2_ adsorption–desorption isotherms and (**b**) pore size distribution profiles of dual-stage char-derived (DSC)-SO_4_ and rising co-current char-derived (RCC)-SO_4_ catalysts.

N_2_ physisorption was used to measure the textural properties of the catalysts and char samples. Figure 1a,b depicts the N_2_ adsorption–desorption isotherms and the pore size spreading profiles of the produced mesoporous DSC-SO_4_ and RCC-SO_4_ catalysts. Typically, the shape of N_2_ physisorption isotherm belongs to the type IV category. As is evident in Figure 1a, the adsorption isotherm of N_2_ is present at a very low pressure (P/P0 < 0.4) range and represents weak adsorption of N_2_ for both catalysts, i.e. mesoporous DSC-SO_4_ and RCC-SO_4_ [18].

Table 1 indicates that the specific surface area and average pore size of DSC and DSC-SO_4_ were 587 m^2^·g^−1^, 3.8 nm, and 527 m^2^·g^−1^, 2.67 nm, respectively. On the other hand, the specific surface area and average pore size of RCC were 419 m^2^·g^−1^ and 3.7 nm, respectively, and those of RCC-SO_4_ were 348 m^2^·g^−1^ and 3.2 nm, respectively. The findings revealed that the acid treatment had a negative impact on the specific surface area and porosity of the synthesized catalysts, and this reduction confirmed that the sulfonic group was impregnated successfully onto the char surface. It is noted that the mesoporous structure of catalysts was well maintained even after the acid treatment. Moreover, another important observation is that a higher surface area of the support provided more space to the sulfonic groups to spread over the mesoporous surface. According to Table 1, the S_BET_ of DSC and DSC-SO_4_ remained higher than that of RCC and RCC-SO_4_. Previously reported data showed that pyrolysis char-based catalysts had a surface area in the range of 20 m^2^·g^−1^ to 250 m^2^·g^−1^ [19,20]. Vittoria et al. [21] employed a dual-stage fixed-bed technology to prepare char materials. According to the reported data, the synthesized material possessed a specific surface area of 297 m^2^·g^−1^, pore size of 4.5 nm, and pore volume of 0.26 cm^3^·g^−1^. Therefore, the DSC and RCC chars used in this work had much larger surface areas and total pore volume even after post-sulfonation, compared with previous materials.

It is important to mention that the DSC and RCC materials had pore diameters of 3.8 nm and 3.7 nm, respectively. However, after the acid treatment, the pore size dropped to 2.6 nm and 3.2 nm for the DSC-SO_4_ and RCC-SO_4_ catalysts, respectively, which shows that the mesoporous structure of DSC-SO_4_ and RCC-SO_4_ catalysts was well preserved even after acid treatment. 

#### 3.1.2. Acid Density Analysis via NH_3_-TPD

**Figure 2:**
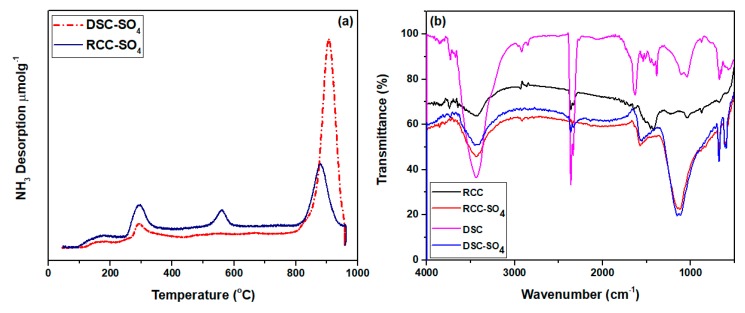
(**a**) NH_3_-TPD profiles of the synthesized mesoporous DSC-SO_4_ and RCC-SO_4_ catalysts and (**b**) FT-IR spectra of DSC, DSC-SO_4_, RCC, and RCC-SO_4_ samples.

The acidic nature of the sulfonated char catalysts was measured through the NH_3_-TPD method, as presented in Figure 2a and Table 1. In Figure 2a, three distinct desorption peaks were obtained in the range of 250–350 °C and 850–970 °C, which showed the occurrence of two different kinds of acid sites. The two different types of peaks indicated the occurrence of weak Bronsted acid sites corresponding to lower-temperature peaks, whereas the higher-temperature peak indicated the presence of a strong Bronsted acid site [22]. It was observed that the pristine DSC-SO_4_ catalyst possessed higher acid density (3.38 mmol·g^−1^), whereas, the RCC-SO_4_ catalyst showed lower acid density (2.68 mmol·g^−1^). This shows that the processed dual-stage gasification char possessed a stronger acid site density than the rising co-current gasification char did. This supports the fact that a higher surface area provides more space and chances to sulfonic particles to disperse onto the mesoporous channels [23].

#### 3.1.3. Functional Groups Determination

The functional groups of both mesoporous acidic catalysts were identified using the FT-IR technique. Figure 2b illustrates the FT-IR results of the pristine chars and char-based acid catalysts. The intense stretching mode from 1040 cm^−1^ to 1210 cm^−1^ was attributed to the sulfonic acid groups, since this stretching mode is absent in the spectra of the char samples [24]. This indicates the successful introduction of sulfonic acid groups onto the char surface. The stretching mode bands in the range of 1520–1705 cm^−1^ authenticate the presence of carboxyl groups [24]. The O–H stretching of moisture and traces of amines was also confirmed, since an intense stretching mode of 3400 cm^-1^ was present in the spectra of all samples. Moreover, the presence of the hydroxyl group in the catalysts was also assured by the O–H stretching between 3200 cm^−1^ and 3600 cm^−1^ [25,26]. 

It was observed that the O–H stretching band was decreased after the sulfonation treatment, which established the formation of the SO_3_H bond on the surface of RCC-SO_4_ and DSC-SO_4_ catalysts. It should be noted that very weak O–H stretching mode in the spectra of RCC, RCC-SO_4_, and DSC-SO_4_ catalysts proved the hydrophobic surface of the catalyst.

#### 3.1.4. Morphology Evaluation of DSC-SO_4_ and RCC-SO_4_ Catalysts

**Figure 3:**
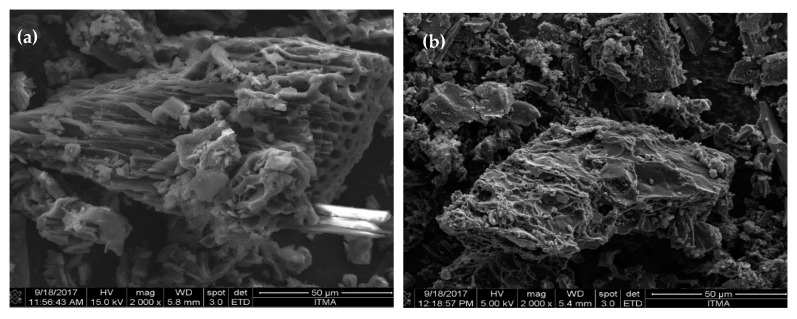
FE-SEM images of the mesoporous (**a**) DSC-SO_4_ and (**b**) RCC-SO_4_ catalysts.

The morphology of the mesoporous acidic DSC-SO_4_ and RCC-SO_4_ catalysts was determined by FE-SEM. As shown in Figure 3, the FE-SEM images confirmed the presence of anomalous pore shapes and micro-channel-like shapes on the surface of the catalysts. These findings are in accord with a previous report [27].

### 3.2. Optimization Study for FAME Production

#### 3.2.1. Influence of Catalyst Concentration

**Figure 4:**
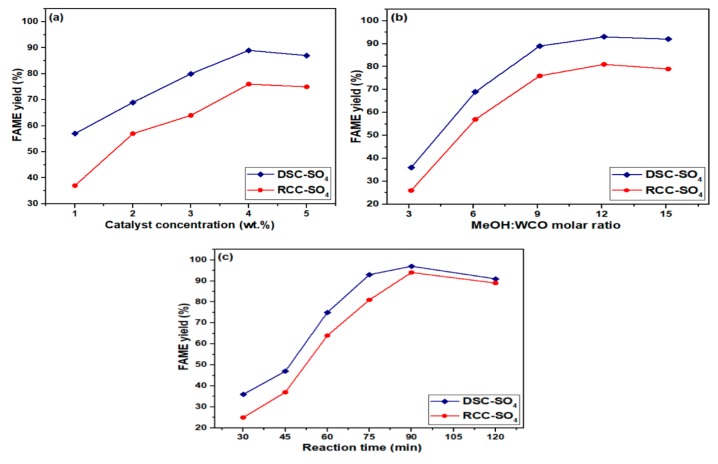
(**a**) Influence of catalyst concentration on fatty acid methyl esters (FAME) conversion; (**b**) influence of MeOH/waste cooking oil (WCO) molar ratio on FAME conversion; (**c**) influence of reaction time on FAME conversion.

The influence of catalyst concentration on FAME production from WCO is illustrated in Figure 4a. The findings suggest that the sulfonated dual-stage gasification char catalyst had a higher FAME yield compared to the sulfonated rising co-current gasification char catalyst, which was due to its higher surface area and total acid density. In general, catalysts with high acid density possess strong catalytic ability. The effect of the amount of catalyst on FAME yield was studied by varying the amount of the catalysts from 1 wt.% to 5 wt.%, while other parameters, such as 9:1 methanol/oil molar ratio, 75 min reaction time, and 65 °C reaction temperature, were kept constant. It was noted that increasing the concentration of the catalyst from 1 to 4 wt.%, increased the FAME yield from 57% to 89%, respectively. However, applying a higher amount of the catalyst caused the FAME yield to start decreasing and limited mass transfer [28]. It was noticed that 4 wt.% of the produced DSC-SO_4_ and RCC-SO_4_ catalysts was the optimum concentration to maximize the contact between the reactant molecules and the catalytically active sites.

#### 3.2.2. Influence of Methanol/Oil Molar Ratio

In this study, the influence of different methanol/oil molar ratios varying from 3:1 to 15:1 was investigated. Figure 4b illustrates that the methanol/oil molar ratio affected the FAME yield. Other transesterification conditions were fixed at 4 wt.% of catalyst, 75 min of reaction time, and 65 °C of reaction temperature. Figure 4b depicts that the FAME yield increased with the increase of the molar ratio from 3:1 to 12:1, whereas a further increase in molar ratio had a negative impact on the FAME yield. This may be attributed to the blockage of the active agent at the active sites [29]. The highest FAME yields of 97% and 89% were attained under the optimal methanol/WCO molar ratio of 12:1 in the presence of mesoporous DSC-SO_4_ and RCC-SO_4_ catalysts, respectively. The DSC-SO_4_ a achieved a higher conversion of FAME compared with the RCC-SO_4_ catalyst. This shows that higher acid density and surface area improved the catalytic activity of the DSC-SO_4_.

#### 3.2.3. Influence of Reaction Time

The influence of reaction times varying from 30 to 150 min was studied for the mesoporous DSC-SO_4_ and RCC-SO_4_ catalysts, while keeping other transesterification conditions fixed as follows: 4 wt.% of catalyst amount, 12:1 of methanol/oil molar ratio, and 65 °C reaction temperature. The reaction time had a prominent effect on FAME conversion. As shown in Figure 4c, in the first 45 min, FAME conversion was insignificant over the mesoporous DSC-SO_4_ and RCC-SO_4_ catalysts. However, by increasing the reaction time to 75 min, the FAME yield increased to 93% and 81% for the mesoporous DSC-SO_4_ and RCC-SO_4_ catalysts, respectively. Both catalysts showed the same trend of FAME yield with respect to time. Figure 4c shows that reaction time and FAME conversion were positively correlated (both quantities increased or decreased simultaneously) throughout the process, reaching maximum values at 90 min and then simultaneously starting to decrease until the reaction time reached 120 min.

It is worth mentioning that a further increase in reaction time can result in the evaporation of methanol and disturb the equilibrium of the reaction [7,30]. The highest WCO methyl FAME yields of 97% and 94% were achieved after 90 min in the presence of the mesoporous DSC-SO_4_ and RCC-SO_4_ catalysts, respectively. According to our results, both selected gasification techniques are promising to activate mesoporous char samples. Although the mesoporous DSC-SO_4_ catalyst possessed better textural properties in terms of surface area and acid density, the mesoporous RCC-SO_4_ catalyst possessed larger pore diameters which allowed the presence of larger reagents into the mesopore channels. This proves that large surface area and high acid density are not the only factors in getting high FAME yields, since a large pore size plays its role as well.

The optimum reaction conditions obtained for the transesterification of WCO were a catalyst amount of 4 wt.%, a methanol/oil molar ratio of 12:1, and an operating time of 90 min under a constant reaction temperature of 65 °C. These optimal conditions resulted in a FAME conversion of 97% and 83% over the mesoporous DSC-SO_4_ and RCC-SO_4_ catalysts, respectively. 

#### 3.2.4. Reusability of DSC-SO_4_ and RCC-SO_4_ Catalysts

**Figure 5:**
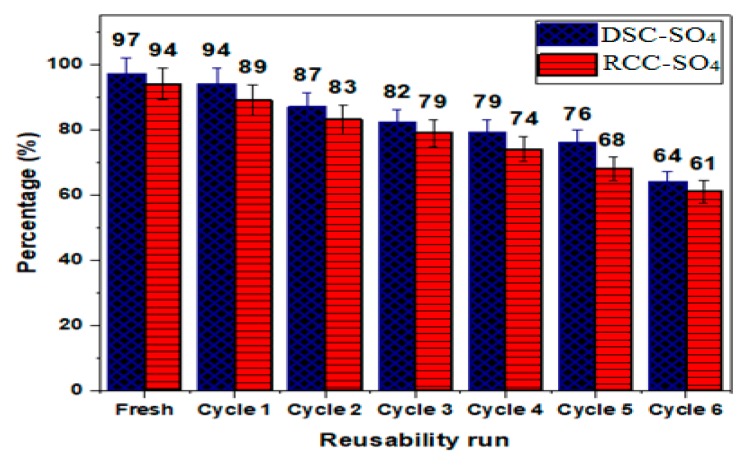
The reusability of the mesoporous DSC-SO_4_ and RCC-SO_4_ catalysts for six reaction runs at optimum reaction conditions.

The catalytic activities of the mesoporous DSC-SO_4_ and RCC-SO_4_ catalysts were investigated in successive runs of transesterification to produce FAME under the optimized reaction conditions. As illustrated in Figure 5, both char-based catalysts gave a significant FAME yield up to the sixth consecutive use without any further treatment. A gradual decrease in FAME yield was observed through six consecutive uses for both mesoporous DSC-SO_4_ and RCC-SO_4_ catalysts, with the FAME yields dropping by 22% and 28%, respectively. The reduction in the FAME yield may be caused by the blockage of the pore channels, which could be due to the leaching of the active sulfonic group. These results show that both mesoporous catalysts (DSC-SO_4_ and RCC-SO_4_) possess high potential as alternative, carbon-based, cheap, and renewable catalysts for esterification reactions and can be used instead of more expensive support materials.

## 4. Conclusions

The sulfonated mesoporous dual-stage gasification char (DSC-SO_4_) and rising co-current gasification char (RCC-SO_4_) catalysts were synthesized by the impregnation method. The synthesized DSC-SO_4_ and RCC-SO_4_ catalysts were applied for biodiesel production through the transesterification of WCO. It was observed that the mesoporous DSC-SO_4_ catalyst had a high specific surface area of 527 m^2^·g^-1^ and yielded 97% of FAME conversion, whereas the RCC-SO_4_ catalyst possessed a specific surface area of 348 m^2^·g^-1^ and provided a FAME conversion of 94%. The highest conversion yield was obtained by using the following optimum conditions: a methanol/oil molar ratio of 12:1, a reaction time of 60 min, and a temperature of 65 °C by employing 4 wt.% of the catalyst. Moreover, the synthesized mesoporous char-based acid catalysts show excellent reusability in the transesterification of WCO for successive six times without using additional treatment, emphasizing the need to use char as a catalyst support instead of disposing of it as waste.

## Figures and Tables

**Table 1:** Textural and physicochemical characteristics of the raw char and synthesized char-based samples.

Catalyst	S_BET_ (m^2^·g^−1^)	D_p_ (nm)	V_p_ (cm^3^·g^−1^)	Acid Density (mmol·g^−1^)
DSC	587 ± 1.71	3.8 ± 0.05	0.30 ± 0.03	1.62
DSC-SO_4_	527 ± 1.25	2.6 ± 0.01	0.35 ± 0.05	3.38
RCC	419 ± 1.43	3.7 ± 0.07	0.37 ± 0.05	1.28
RCC-SO_4_	348 ± 1.76	3.2 ± 0.09	0.32 ± 0.02	2.68
